# Population Genetic Data of 30 Insertion-Deletion Markers in the Polish Population

**DOI:** 10.3390/genes13101683

**Published:** 2022-09-20

**Authors:** Monica Abreu-Glowacka, Witold Pepinski, Eliza Michalak, Magdalena Konarzewska, Krzysztof Zak, Malgorzata Skawronska, Anna Niemcunowicz-Janica, Ireneusz Soltyszewski, Pawel Krajewski, Czeslaw Zaba

**Affiliations:** 1 Department of Forensic Medicine, Poznan University of Medical Sciences, 60-781 Poznan, Poland; 2 Department of Forensic Medicine, Medical University of Bialystok, 15-269 Bialystok, Poland; 3 Department of Forensic Medicine, Medical University of Warsaw, 02-007 Warsaw, Poland

**Keywords:** Poland, population, InDels, Investigator DIPplex, forensic genetics

## Abstract

(1) Background: Insertion-deletion (InDel) markers show the advantages of both short tandem repeats (STRs) and single nucleotide polymorphisms (SNPs) and are considered alternative markers in forensic genetics. (2) Methods: Allelic frequencies and corresponding forensic efficiency parameters of 30 autosomal polymorphic InDel loci included in the Investigator DIPplex kit (Qiagen) were obtained in a sample of 631 unrelated Polish individuals. Allelic frequency data were compared with those reported for selected populations (3) Results: All the loci conformed with Hardy-Weinberg equilibrium after applying a Bonferroni correction and no pair-wise significant linkage disequilibrium was detected. (4) Conclusions: DIPplex Kit differences were high among populations worldwide. The InDel markers are highly discriminating for human identification purposes in the Polish population.

## 1. Introduction

Insertion-deletion (InDel) diallelic polymorphisms are spread in the human genome on all 24 chromosomes (approximately one InDel per 7.2 kbps) and result from insertion and/or deletion of short sequences of 1 to 10,000 bps in length [1]. Due to amplicon sizes designed to be short (50–160 bps), relatively low mutation rates (less than 1 × 10^−8^), absence of microvariant products and stutter peaks, and automated typing using capillary electrophoresis-based instruments InDel markers have received special attention in forensic genetics practice as alternative markers to Short Tandem Repeats (STRs) and Single Nucleotide Polymorphisms (SNPs) [2,3,4]. In addition to forensic DNA casework, InDels exhibiting large differences in allelic frequencies among different ancestral groups or geographically distant populations may serve as ancestry-informative markers (AIMs) to ascertain population substructure and predict biogeographical origin [5]. The Investigator DIPplex kit (Qiagen) contains 30 forensic-related InDel loci and a sex marker–amelogenin for the simultaneous PCR amplification. Numerous Investigator DIPplex datasets have been reported for mainly Asian and European populations, however, studies on other population data for these markers are still limited.

Poland is located in central Europe at latitude 51.919438 and longitude 19.145136. Based on the allochthonous theory, Poles descended from Western Slavs from the Upper Dnieper basin which expanded to the region between Rivers Warta (Varta) and Wisla (Vistula) in the 5th or 6th century [6]. Since the early Middle Ages, the country has been invaded successively by Germans, Balts, and Mongols, yet sustained its national integrity. From 1772 to 1918 the country was partitioned by the empires of Russia, Prussia, and Austria. Before World War II Poland was inhabited by a variety of ethnic communities including Germans, Ukrainians, and Yiddish-speaking Jews. The official figure of Polish war losses issued in 1947 was 6,028,000 and referred exclusively to losses within the post-war frontiers. The post-war period starting in 1946 witnessed intense demographic processes and an unprecedented birth rate resulting in the number of inhabitants increasing the number by ca. 14 million until 1988. Since then, the natural increase rate (balance of births against deaths) has neared nil [6]. According to UN estimates the population in Poland was expected to reach 39,857,145 by 1 July 2022 [7]. The observed genetic homogeneity within Poland, accompanied by minor differences at the regional level, is most probably due to a potentially homogeneous population of ancestral Slavs, a substantial loss of both major and minor ethnic communities from the country’s territory, and/or the forced displacement, expulsion, and deportation during and soon after WWII. Currently, around 97% of the population claim sole or partial Polish nationality with only 450,000 members of ethnic groups of non-Polish ancestry, including Belarusian, Czech, German, Lithuanian, Russian, Slovak, and Ukrainian minorities settled nearby Poland’s borders as a result of population displacements from bordering pre-war areas [8]. Furthermore, not until the collapse of communism in 1989, was minority ethnic identity cultivated officially. Previous population genetic studies confirmed that the Polish population is homogenous in terms of autosomal STR and mtDNA polymorphisms [9,10]. Also, studies on Y chromosome Y-SNP and Y-STR distributions indicated that paternal lineages are homogenous within Poland and distinct from the patrilineages in the neighboring populations [11,12,13].

The aim of our study was to provide reference allelic frequencies of 30 autosomal InDels for the Polish population sample and to calculate forensic efficiency parameters to be used in forensic genetics practice. We were also interested in whether between-population differences can be detected using the Indel set based on our results and selected published data.

## 2. Material and Methods

### 2.1. Sampling

Buccal swabs were collected from 631 unrelated healthy Polish individuals (319 males and 312 females) living in Poznan, Warsaw, and Bialystok regions. DNA samples were extracted using the QIAamp DNA Mini (Qiagen, Hilden, Germany) and quantified using the Quantifiler Human DNA Quantification Kit on a 7500 Real-Time PCR instrument (Thermo Fisher Scientific, Waltham, MO, USA). Sample concentrations were adjusted to 0.5 ng/μL according to the manufacturer’s recommendations.

### 2.2. PCR Amplification and InDel Genotyping

About 0.5 ng genomic DNA templates were amplified in a 25 μL reaction volume on a GeneAmp PCR system 9700 (Thermo Fisher Scientific, Waltham, MO, USA), following the kit manufacturer’s manual. The PCR products were separated and detected on the 3500 Genetic Analyzer (Thermo Fisher Scientific, Waltham, MO, USA). The SST-BTO size standard and reference allelic ladder provided in the kit were used for data analysis and genotyping by GeneMapper ID-X v.1.5 software (Thermo Fisher Scientific, Waltham, MO, USA). The experiments were carried out using the 9948 male DNA positive control and the negative control of ddH2O. The recommendations of the DNA Commission of the International Society for Forensic Genetics (ISFG) on the analysis of forensic markers [14] and internal quality control requirements according to the ISO 17025 standard were strictly followed. The HLD (human locus deletion/insertion polymorphism) numbers were used to designate Indel loci in the DIPplex kit. The corresponding RefSNP (rs) numbers are listed in Table 1.

### 2.3. Statistical Analyses

An online tool for STR Analysis for Forensics (STRAF v.2.0.8) [15] was employed to calculate allele frequencies of the 30 markers, genetic diversity parameters, and corresponding forensic genetic parameters: Polymorphism Information Content (PIC), Power of Discrimination (PD), Power of Exclusion (PE), Typical Paternity Index (TPI), and also to test for deviations from HWE (10,000 permutations) and Linkage Disequilibrium (LD). The possible LD between three common STR and DIPplex locus pairs mapped on the same chromosome arms (5q, 7q, 8q) was estimated via likelihood ratio tests implemented in Arlequin v.3.5 [16]. The significance level for multiple testing was adjusted using the Bonferroni correction. The POPTREE2 software [17] was used to calculate sample bias corrected interpopulation FST distances [18] and also to reconstruct the neighbor-joining (NJ) phylogenetic tree [19] based on allelic frequency data. Twenty populations were included in the FST calculation: Poland (this study), Spain [20], Mexico (Chihuahua) [21], South Korea [22], Finland, Somalia [23], Pakistan (Punjab) [24], South Africa Afrikaner, Zulu) [25], Iraq, Lithuania, Slovenia, Turkey [26], Vietnam, Nigeria [27], Bahrain [28], immigrants from Angola and Mozambique in Lisbon, Portugal [29], Brazil [30], and China (Han) [31]. A multidimensional scaling analysis (MDS) plot on the pairwise FST distances was drawn using the SMACOF approach [32] in the R package v.4.1.1 [33].

## 3. Results

The allele frequency distributions and corresponding forensic efficiency parameters based on the raw genotypes (submitted in Supplementary Table S1) are shown in Table 1. Insertion allelic frequencies (DIP+) of 30 markers range from 0.3487 for HLD114 to 0.6648 for HLD56. After applying the Bonferroni correction of multiple comparisons (0.05/30 = 0.00167), no deviations from HWE were observed (0.0157 < *p* < 1.0000) with the lowest *p-*value at HLD93 locus. No significant LD (*p* > 0.000115) was observed between the pairwise InDels after applying the Bonferroni adjustment (0.05/435), which indicates random association among the 30 InDel loci in the studied population. Moreover, no significant linkage was detected (*p* > 0.0190) between the three most likely STR candidates and DIPplex locus pairs (CSF1PO-HLD67, D7S820-HLD81, and D8S1179-HLD84) located on the same chromosomes. The Ho and He values varied from 0.4460 (HLD56) to 0.5004 (HLD111), and from 0.4263 (HLD39) to 0.5436 (HLD93), with the averages of 0.4764 and 0.4896, respectively. The least polymorphic locus is HLD56 with a PIC of 0.3464, while the most informative locus is HLD111 with a PIC of 0.3750. The PD and PE values range from 0.5949 (HLD93) to 0.6422 (HLD6) and from 0.1307 (HLD39) to 0.2286 (HLD93), respectively. TPI values vary from 0.8715 (HLD39) to 1.0955 (HLD93). The combined Probability of Match (cPM) is 1.7392 × 10^−13^. The combined Power of Discrimination (cPD) is >0.9999 and the combined Power of Exclusion (cPE) is 0.9961. To evaluate whether the representative regions of Poland are homogenous with respect to the studied InDels, locus-by-locus Analysis of Molecular Variance (AMOVA) was performed. No statistically significant variation caused by differences among population samples of respective regions was detected (0.1009 < *p* < 0.9567) which indicates that all of the variances were found within the regional Polish populations.

## 4. Discussion

The single locus parameters and the cumulative forensic efficiency indexes calculated in this study indicate that this panel is informative in the Polish population and can be useful for forensic individual identification. As it has been shown previously [27,34] based on the calculated TPI value the Investigator DIPplex kit is not sufficient as a stand-alone system in paternity tests, however, due to their reduced mutation rates [35]. InDels may serve as an extension to STR platforms in deficient or inconclusive cases [36,37]. As estimated by Krawczak, at least 60 maximally informative SNPs would be required to yield the same power of paternity exclusion as the set of 14 microsatellites of the average allele number 9.5 and the average gene diversity 0.77, since the gene diversity of an SNP will normally be smaller than 0.5% [38].

**Figure 1:**
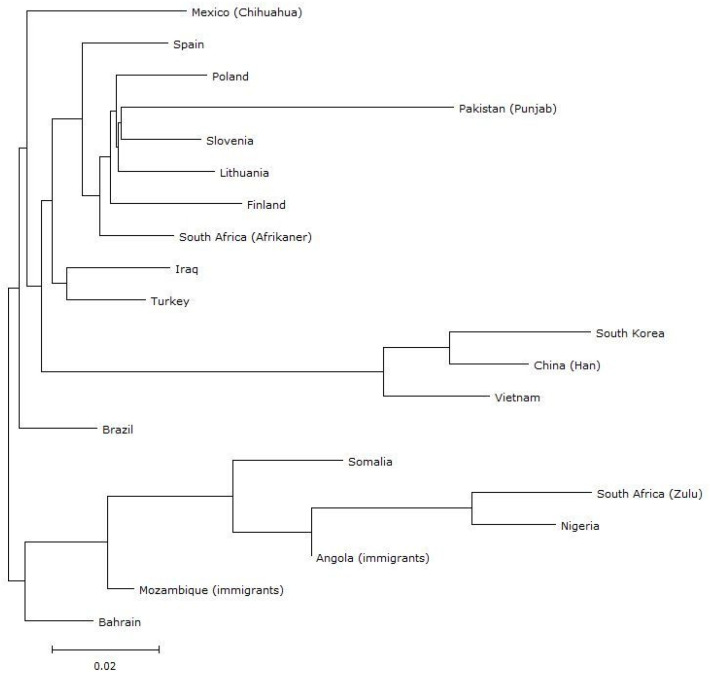
Phylogenetic tree (NJ) of the Polish population and 19 other reference populations based on FST distances.

**Figure 2:**
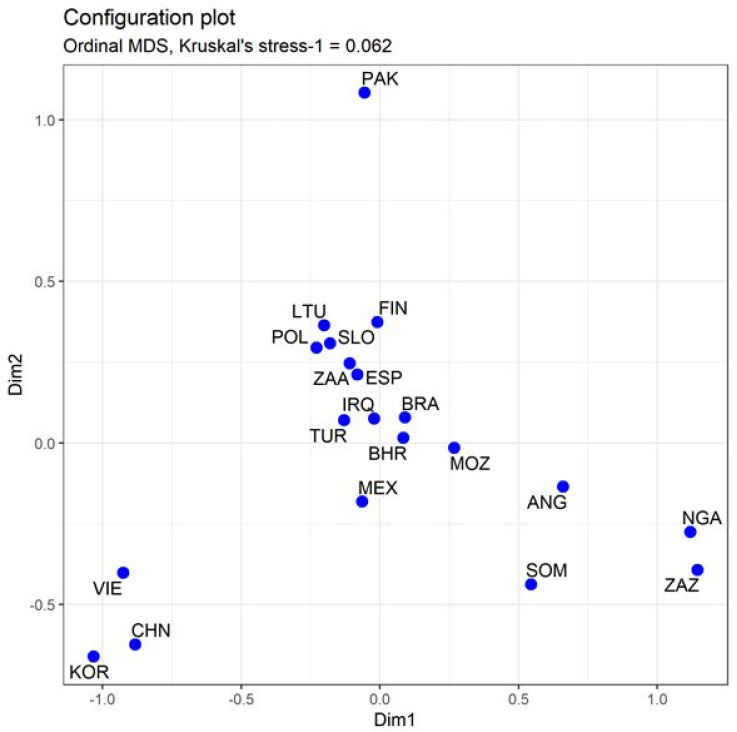
Multi-dimensional scale (MDS) plot of the Polish population and 19 other reference populations based on FST distances.

Genetic distance is an important indicator of relatedness among populations. The fixation index (FST) is a comparative measure of genetic variation in a population due to genetic structure or differentiation between populations. To compare the Polish population with the 19 previously investigated populations, sample bias corrected FST distances were calculated among all pairs of populations based on allelic frequencies of the 30 InDel loci (Table 2). In general, higher FST values represent more genetic differentiation between two populations. Large genetic distances were found between the Polish population and Somalians, Nigerians, South African Zulus, South Koreans, Vietnamese, and Chinese. We then reconstructed phylogenetic relationships on the basis of FST genetic distances (Figure 1). As is shown in the graphic representation of these distances, the populations in our study are grouped in separate branches according to continental or regional biogeographical ancestry. Among the other populations, Poles share the most genetic relatedness with Slovenians and Lithuanians, followed by Finns and Spanish in the same cluster. Black African and East Asian populations cluster in two different genetic structures distant from European populations and Pakistanis are found on a separate branch. To further investigate genetic relationships between Poles and worldwide populations an MDS plot was drawn from the FST values to represent genetic relationships between the populations in multidimensional space. As shown in Figure 2, East Asians, Pakistanis, and most Black Africans are allocated apart on the bottom left, upper, and bottom right of the plot, respectively, thus can be clearly distinguished from the other groups. Europeans are found in the middle of the plot. The other populations are distributed between the Europeans and Africans. South African Afrikaners tend to be in a close relationship with Europeans, which may be due to their descent from predominantly Dutch settlers in the 17th and 18th centuries.

Most of the evaluated Indels show Ho of 0.5000 approx., which makes them suitable for forensic human identification as identity-informative markers. On the other hand, markers that exhibit low heterozygosity and different allelic frequency distributions between populations (high individual locus-specific FST) may be potentially used as ancestry-informative (AIM-InDels) in distinguishing between populations of interest. Therefore, three candidates for AIM-InDels are likely in our batch: HLD111, HLD118, and HLD81 of FST = 0.2607, 0.2781, and 0.2221, respectively. Thus, due to increased interest, enhanced sets of more effective AIMs are needed for commercial development and validation to identify ancestry contributions of admixtures.

## 5. Conclusions

We provided the first comprehensive analysis of DIPplex Kit markers for the Polish population with details to calculate forensic efficiency parameters and investigate genetic diversity.

Based on interpopulation comparisons the 30 InDel differences are high enough to perform intercontinental forensic population analysis.

Our findings indicate that the DIPplex Kit can be used in forensic applications in the Polish population to increase the power of evidence of the conventional STR markers.

## Figures and Tables

**Table 1:** Allelic frequencies and forensic efficiency parameters for 30 InDels in the Polish population sample (*n* = 631).

HLD	Chromosomal	GenBank	DIP(−)	DIP(+)	Ho	He	p HWE	PIC	PD	PE	TPI
	Location	SNP ID									
HLD77	7q31.1	rs1611048	0.4422	0.5578	0.4937	0.4628	0.1224	0.3716	0.6348	0.1569	0.9307
HLD45	2q31.1	rs2307959	0.4739	0.5261	0.4990	0.5040	0.8096	0.3743	0.6216	0.1910	1.0080
HLD131	7q36.2	rs1611001	0.4564	0.5436	0.4966	0.4628	0.0934	0.3731	0.6377	0.1569	0.9307
HLD70	6q16.1	rs2307652	0.4731	0.5269	0.4989	0.4929	0.8100	0.3743	0.6270	0.1813	0.9859
HLD6	16q13	rs1610905	0.4707	0.5293	0.4987	0.4564	0.0384	0.3741	0.6422	0.1521	0.9198
HLD111	17p11.2	rs1305047	0.4976	0.5024	0.5004	0.4818	0.3837	0.3750	0.6336	0.1720	0.9648
HLD58	5q14.1	rs1610937	0.4461	0.5539	0.4946	0.4739	0.2955	0.3721	0.6312	0.1656	0.9503
HLD56	4q25	rs2308292	0.3352	0.6648	0.4460	0.4358	0.5868	0.3464	0.5966	0.1372	0.8862
HLD118	20p11.1	rs16438	0.5143	0.4857	0.5000	0.4707	0.1451	0.3748	0.6380	0.1631	0.9446
HLD92	11q22.2	rs17174476	0.5919	0.4081	0.4835	0.4707	0.5146	0.3664	0.6215	0.1631	0.9446
HLD93	12q22	rs2307570	0.4477	0.5523	0.4949	0.5436	0.0157	0.3722	0.5949	0.2286	1.0955
HLD99	14q23.1	rs2308163	0.4097	0.5903	0.4841	0.4929	0.6853	0.3667	0.6122	0.1813	0.9859
HLD88	9q22.32	rs8190570	0.5895	0.4105	0.4843	0.4881	0.8711	0.3669	0.6147	0.1773	0.9768
HLD101	15q26.1	rs2307433	0.4786	0.5214	0.4995	0.4913	0.7021	0.3745	0.6283	0.1800	0.9829
HLD67	5q33.2	rs1305056	0.3954	0.6046	0.4785	0.4834	0.8013	0.3638	0.6110	0.1734	0.9678
HLD83	8p22	rs2308072	0.5127	0.4873	0.5001	0.5119	0.5797	0.3748	0.6185	0.1981	1.0244
HLD114	17p13.3	rs2307581	0.6513	0.3487	0.4545	0.4469	0.7246	0.3510	0.6015	0.1451	0.9040
HLD48	2q11.2	rs28369942	0.4770	0.5230	0.4993	0.4628	0.0669	0.3745	0.6405	0.1569	0.9307
HLD124	22q12.3	rs6481	0.3574	0.6426	0.4597	0.4612	1.0000	0.3538	0.6015	0.1557	0.9279
HLD122	21q22.11	rs8178524	0.5388	0.4612	0.4974	0.5261	0.1444	0.3735	0.6079	0.2114	1.0552
HLD125	22q11.23	rs16388	0.4889	0.5111	0.5002	0.4897	0.6298	0.3749	0.6297	0.1786	0.9798
HLD64	5q12.3	rs1610935	0.4532	0.5468	0.4960	0.4501	0.0258	0.3728	0.6419	0.1474	0.9092
HLD81	7q21.3	rs17879936	0.5689	0.4311	0.4909	0.4437	0.0184	0.3702	0.6389	0.1428	0.8989
HLD136	22q13.1	rs16363	0.5475	0.4525	0.4959	0.4834	0.5776	0.3727	0.6284	0.1734	0.9678
HLD133	3p22.1	rs2067235	0.4461	0.5539	0.4946	0.4739	0.2933	0.3721	0.6312	0.1656	0.9503
HLD97	13q12.3	rs17238892	0.4493	0.5507	0.4952	0.4834	0.5686	0.3724	0.6278	0.1734	0.9678
HLD40	1p32.3	rs2307956	0.5634	0.4366	0.4924	0.4580	0.0871	0.3709	0.6353	0.1533	0.9225
HLD128	1q31.3	rs2307924	0.5563	0.4437	0.4941	0.4691	0.2199	0.3718	0.6327	0.1619	0.9418
HLD39	1p22.1	rs17878444	0.6268	0.3732	0.4682	0.4263	0.0265	0.3584	0.6216	0.1307	0.8715
HLD84	8q24.12	rs3081400	0.4532	0.5468	0.4960	0.4945	1.0000	0.3728	0.6234	0.1827	0.9890

Legend: DIP(−) frequency of deletion allele; DIP(+) frequency of insertion allele; Ho observed heterozygosity; He expected heterozygosity; p HWE probability values for Hardy-Weinberg equilibrium test; PIC Polymorphism Information Content; PD Power of Discrimination; PE Power of Exclusion; TPI Typical Paternity Index.

**Table 2:** Matrix of the pairwise FST genetic distances among the Polish population and 19 other reference populations.

	ESP	MEX	KOR	FIN	SOM	PAK	ZAZ	IRQ	LTU	SLO	TUR	VIE	NGA	BHR	ZAA	ANG	MOZ	BRA	CHN
POL	0.037	0.064	0.124	0.044	0.107	0.079	0.163	0.049	0.036	0.033	0.045	0.106	0.155	0.053	0.035	0.098	0.063	0.050	0.116
ESP	*	0.056	0.130	0.047	0.090	0.090	0.144	0.043	0.039	0.038	0.039	0.108	0.135	0.043	0.034	0.082	0.051	0.043	0.122
MEX	+	*	0.129	0.062	0.098	0.127	0.142	0.056	0.064	0.063	0.050	0.116	0.128	0.052	0.057	0.085	0.059	0.045	0.120
KOR	+	+	*	0.136	0.166	0.193	0.224	0.135	0.140	0.127	0.122	0.059	0.227	0.125	0.132	0.172	0.135	0.125	0.041
FIN	+	+	+	*	0.103	0.090	0.147	0.056	0.044	0.041	0.054	0.121	0.137	0.058	0.042	0.088	0.056	0.046	0.125
SOM	+	+	+	+	*	0.149	0.071	0.084	0.108	0.106	0.088	0.138	0.067	0.070	0.098	0.050	0.059	0.073	0.146
PAK	+	+	+	+	+	*	0.198	0.105	0.081	0.078	0.103	0.176	0.192	0.111	0.077	0.137	0.107	0.099	0.178
ZAZ	+	+	+	+	+	+	*	0.132	0.166	0.161	0.147	0.213	0.038	0.107	0.148	0.052	0.076	0.109	0.214
IRQ	+	+	+	+	+	+	+	*	0.051	0.048	0.034	0.112	0.120	0.037	0.045	0.073	0.050	0.045	0.122
LTU		+	+	+	+	+	+	+	*	0.034	0.048	0.116	0.157	0.057	0.037	0.102	0.065	0.051	0.125
SLO	+	+	+	+	+	+	+	+		*	0.044	0.108	0.151	0.052	0.033	0.096	0.061	0.048	0.117
TUR	+	+	+	+	+	+	+		+	+	*	0.098	0.137	0.038	0.040	0.084	0.054	0.045	0.109
VIE	+	+	+	+	+	+	+	+	+	+	+	*	0.214	0.107	0.109	0.158	0.119	0.109	0.047
NGA	+	+	+	+	+	+	+	+	+	+	+	+	*	0.100	0.141	0.043	0.071	0.101	0.217
BHR	+	+	+	+	+	+	+	+	+	+	+	+	+	*	0.046	0.060	0.039	0.039	0.116
ZAA		+	+	+	+	+	+	+	+	+	+	+	+	+	*	0.087	0.054	0.042	0.119
ANG	+	+	+	+	+	+	+	+	+	+	+	+	+	+	+	*	0.042	0.060	0.162
MOZ	+	+	+	+	+	+	+	+	+	+	+	+	+	+	+	+	*	0.039	0.125
BRA	+	+	+	+	+	+	+	+	+	+	+	+	+		+	+	+	*	0.115

Legend: POL Poland; ESP Spain; MEX Mexico (Chihuahua); KOR South Korea; FIN Finland; SOM Somalia; PAK Pakistan (Punjab); ZAZ South Africa (Zulu); IRQ Iraq; LTU Lithuania; SLO Slovenia; TUR Turkey; VIE Vietnam; NGA Nigeria; BHR Bahrain; ZAA South Africa (Afrikaner); ANG Angola (immigrants); MOZ Mozambique (immigrants); BRA Brazil; CHN China (Han). Above diagonal: FST values; below diagonal: corresponding *p* values (*p* < 0.05) (‘+’ denotes statistically significant result). Asterisks * are only used as the content separator.

## Data Availability

Not applicable.

## Supplementary Materials

The following supporting information can be downloaded at: https://www.mdpi.com/article/10.3390/genes13101683/s1 Table S1: Raw genotype data of 30 InDels in the Polish population (*n* = 631).
